# Characteristics and quality of life of people living with comorbid disorders in substance use recovery residences

**DOI:** 10.3389/fpubh.2024.1412934

**Published:** 2024-11-19

**Authors:** Elizabeth O. Obekpa, Sheryl A. McCurdy, Kathryn R. Gallardo, Serena A. Rodriguez, Cecilia Ganduglia Cazaban, H. Shelton Brown, James J. Yang, J. Michael Wilkerson

**Affiliations:** ^1^UTHealth Houston School of Public Health, Institute of Implementation Science, Houston, TX, United States; ^2^Department of Health Promotion and Behavioral Sciences, UTHealth Houston School of Public Health, Houston, TX, United States; ^3^Department of Management, Policy, and Community Health, UTHealth Houston School of Public Health, Houston, TX, United States; ^4^Department of Management, Policy, and Community Health, The University of Texas School of Public Health, Austin, TX, United States; ^5^Department of Biostatistics and Data Science, UTHealth Houston School of Public Health, Houston, TX, United States

**Keywords:** health-related quality of life, EQ-5D-5L, comorbidity, opioid use disorder, medication for opioid use disorder, recovery residences, integrated care, recovery housing

## Abstract

**Background:**

Opioid use disorder (OUD) is associated with significant morbidity and mortality; however, research on physical and mental health comorbidities and health-related quality of life (HRQoL) among people taking medication for OUD (MOUD) and living in recovery residences is sparse. We investigated the prevalence of comorbidities and examined which EQ-5D-5L HRQoL dimensions are most affected by these comorbidities.

**Methods:**

Data were collected from 358 residents living in 14 Texas-based recovery residences from April 2021 to June 2023. The EQ-5D-5L descriptive system comprises five dimensions (mobility, self-care, usual activities, pain/discomfort, anxiety/depression). Each dimension has five levels of perceived problems, dichotomized into “No problems” (level 1) and “Any problems” (levels 2–5) for analyses. Cross-sectional analyses of residents' characteristics, comorbidities (categorized as mental health disorders or association with major body systems), and EQ-5D-5L dimensions were conducted using Chi-squared or Student *t*-tests. Multivariable logistic regression models were used to estimate the odds ratios (ORs) and 95% confidence intervals (CIs).

**Results:**

The mean [SD] age of residents was 36.0 [8.9]. Most residents were non-Hispanic White (68.7%), male (59.7%), unemployed (66.3%), and engaged in polysubstance use (75.4%). The most frequently reported comorbidities were mental health (26.5%), respiratory (26.3%), neurological (19.3%), cardiovascular (18.2%), and musculoskeletal (17.0%) disorders. The most reported HRQoL problems were anxiety/depression (75.8%) and pain/discomfort (53.2%). In the unadjusted regression models, all comorbidities, except mental health (negative association) and digestive (no association) disorders, were positively associated with HRQoL problems. The usual activities dimension was the most affected by comorbidities, followed by mobility and pain/discomfort. Increasing age was positively associated with cardiovascular disorders (aOR = 1.06; 95% CI = 1.03–1.10), musculoskeletal disorders (aOR = 1.03; 95% CI = 1.00–1.06), mobility problems (aOR = 1.05; 95% CI = 1.01–1.09), and pain/discomfort problems (aOR = 1.02; 95% CI = 1.00–1.05). Illicit drug use was positively associated with mobility problems (aOR = 3.36; 95% CI = 1.20–9.45). Neurological (aOR = 2.71; 95% CI = 1.38–5.33) and musculoskeletal (aOR = 2.57; 95% CI = 1.25–5.29) disorders were positively associated with pain/discomfort problems. MOUD duration was negatively associated with mental health disorders (aOR = 0.14; 95% CI = 0.08–0.22) but not HRQoL.

**Conclusions:**

Comorbidities significantly predict HRQoL among individuals with OUD. Our findings highlight the need for an integrated care model to treat OUD and comorbidities to sustain recovery and improve health and HRQoL.

## 1 Introduction

Opioid overdose-related deaths are rising in the United States (US), posing a significant public health crisis, with Opioid use disorder (OUD) having the highest disease burden of any substance use disorder (SUD) (1–3). The interest in perceived health-related quality of life (HRQoL) as a SUD treatment and recovery outcome is growing (4–6). HRQoL encompasses an individual's perception of their health status, including their physical, psychological, social functioning, and wellbeing, and is an important indicator of treatment effectiveness and recovery outcomes (7–11).

The risk factors associated with OUD, including mental and physical comorbidities and polysubstance use, are complex and may involve reverse causality (12, 13). OUD is frequently complicated by, and can also exacerbate, mental and physical comorbidities, including depression, anxiety, chronic pain, bone and mobility conditions, and cardiovascular disorders (12, 14–22). Research has demonstrated that individuals with SUDs are disproportionately affected by comorbid disorders than those without SUDs (20, 23, 24), with over 90% of opioid-related hospitalizations attributed to comorbidities (25). Individuals with OUD and comorbidities have a significantly greater mortality risk than those without OUD and comorbidities (26).

The diagnosis and treatment of comorbid SUD and mental and physical health disorders are intricate, with polysubstance use further complicating care (20) and increasing one's risk of poor health outcomes, including diminished HRQoL and response to treatment, disability, and alcohol- and substance use-related early mortality (27–30). Opioid-related overdose deaths involving benzodiazepines have steadily increased since 2019, with over 12,000 deaths reported in 2021, while deaths involving antidepressants rose from 1,749 in 1999 to over 5,800 in 2021 (31).

The high disease burden and mortality rates call for the integration of OUD treatment, recovery support, and primary care for the concurrent management of OUD and mental and physical health disorders. Research shows that most psychiatric comorbidities in individuals with SUDs improved following SUD treatment integration with primary care (32–34), and integrated healthcare enhanced health outcomes, treatment initiation and adherence rates, and sustained viral response for individuals with comorbid substance use, psychiatric disorders, HIV, and Hepatitis C (35). While primary care settings provide effective, albeit fragmented, settings for SUDs and comorbidity management, recovery residences —sober and safe living communities —may serve as ideal settings for an integrative care approach.

Recovery housing is an effective intervention to support individuals in achieving and maintaining their recovery from SUD, resulting in positive outcomes, such as substance use abstinence, employment, and lower incarceration rates (36–38). However, recovery housing is an underutilized service modality (39) that provides a unique opportunity to increase access to recovery support services and integrated treatment for OUD and comorbidities to improve recovery and health outcomes, including HRQoL. Recovery residences can provide an access point for the identification and treatment of mental and physical comorbidities.

Despite their potential, little research has explored how recovery residences can be used in this manner to support SUD recovery. Furthermore, although the associations between mental disorders and HRQoL among individuals with SUDs are well documented, the literature on HRQOL as an OUD treatment and recovery outcome and examinations of the associations between physical comorbidities and HRQoL is limited (4, 5, 19, 30), particularly among individuals taking MOUD and selecting into recovery residences.

To address this literature gap, we investigated the prevalence of self-reported mental and physical comorbidities among individuals taking MOUD and living in recovery residences. We also identified the predictors of comorbidities and HRQoL across five EQ-5D-5L health dimensions (mobility, self-care, usual activities, pain/discomfort, and anxiety/depression). Finally, we examined the associations between mental and physical comorbidities and the HRQoL dimensions.

## 2 Methods

### 2.1 Study design

Most recovery residences are certified by the National Alliance for Recovery Residences (NARR) affiliates as levels 1–4, with level 4 recovery residences providing the most intense level of care and level 1 recovery residences providing the least level of care (40). The Housing for Opioid Medication-Assisted Recovery Expanded Services (Project HOMES) is an evaluation study being implemented in levels II (monitored) and III (supervised) recovery residences for individuals taking MOUD and residing in five Texas cities (Austin, El Paso, Houston, Midland, and San Angelo). Levels II and III Recovery residences include non-clinical staff who are also in recovery and provide recovery support. Recovery residence operators screened study participants for eligibility in Project HOMES using these inclusion criteria: age 18 or over, currently taking or willing to take MOUD and being able to pay for it, and a commitment to recovery. Written informed consent was obtained, and data collection began 8–14 days after the residents moved into the residences and decided to remain in the program. Residents received a $25 gift card for their time. Data analyzed were collected from April 2021 to June 2023. The institutional review board of the authors' home institution approved the study protocol.

### 2.2 Measurements

#### 2.2.1 Outcome measures—EQ-5D-5L HRQoL dimensions

The EQ-5D-5L instrument measures HRQoL in five dimensions—mobility, self-care, usual activities, pain/discomfort, and anxiety/depression, each with five levels of severity: no problems, slight problems, moderate problems, severe problems, and unable to/extreme problems. Residents were asked to select their health state in a given dimension (Supplementary Table 1). For analyses, responses were dichotomized as “no problems” (level 1) and “any problems” (levels 2, 3, 4, and 5) to describe the frequency of problems across each dimension and identify the health dimensions most impacted by comorbidities (41).

#### 2.2.2 Sociodemographic characteristics

Residents' sociodemographic characteristics include age and categorical measures of sex at birth, race-ethnicity, education, employment, and marital status (42).

#### 2.2.3 Satisfaction with health

Residents were asked, “How satisfied are you with your health? “Responses were on a 5-point Likert-type scale, ranging from 0 ‘Very dissatisfied' to 4 ‘Very satisfied' (42).

#### 2.2.4 Duration of MOUD

Residents were asked, “What date did you first start your current MOUD prescription? For analyses, responses were converted to years on MOUD and categorized as < 1 year, 1 to 5 years, and >5 years.

#### 2.2.5 Comorbid disorders

Residents were asked to select all medical diagnoses from a list. Each comorbidity was classified based on its association with seven major body systems (respiratory, neurological, cardiovascular, musculoskeletal, digestive, endocrine, and urogenital) and mental health disorders. Due to the small cell count, several diseases, including HIV and Hepatitis A, B, and C, were categorized as other comorbid disorders. For analyses, each comorbid body system disorder was dichotomized and categorized as “No” and “Yes” (Supplementary Table 2).

#### 2.2.6 Alcohol, tobacco, and substance use

Hazardous drinking was assessed using the 10-item Alcohol Use Disorders Identification Test (AUDIT) (43). The AUDIT was scored on a scale of 0–4 and summed to give a range of scores from 0 to 40 (43). A score of 0–7 indicates no or low risk for hazardous drinking (43). Residents with scores of 8 or more had a high risk for hazardous drinking (43). The Cronbach's alpha coefficient of the AUDIT in our study was 0.88, indicating good internal consistency (44).

Residents were asked to select substances they had used in the past 3 months from a list that included illicit substances, non-medical use of marijuana, and the misuse of prescription medications, such as opioids, stimulants, sedatives or sleeping pills, and barbiturates (42). Misuse was defined as using a medication without a prescription or taking it in higher doses, more frequently, or for longer than directed. The use of more than one substance (excluding alcohol and tobacco) was categorized as polysubstance use. The most frequently reported substances used were alcohol (96.1%), tobacco (91.9%), street opioids (55.0%), including heroin and fentanyl, methamphetamine (42.2%), benzodiazepines (38.8%), marijuana (37.5%), prescription opioids (26.0%), including morphine and methadone, and cocaine (24.3%) (Supplementary Table 3).

### 2.3 Statistical analyses

We collected data from residents taking MOUD after their entry into 14 Texas-based recovery residences to estimate the prevalence of mental and physical comorbidities among residents and their association with EQ-5D-5L dimensions. Continuous variables were described by means and standard deviations (SD), while categorical variables were described by numbers and percentages. Chi-Square, Fisher's exact, or Student *T*-tests were used to examine the bivariate associations between residents' sociodemographic characteristics and EQ-5D-5L dimensions (Table 1), comorbid disorders and EQ-5D-5L dimensions (Table 2), and residents' characteristics and comorbid disorders (Table 3). Statistically significant variables (*p* ≤ 0.05) from these tests were then entered into univariate and multivariable logistic regression models to estimate the unadjusted and adjusted odds ratios (ORs and aORs) and 95% confidence intervals (CIs) to predict residents' characteristics associated with comorbid disorders (Table 4) and comorbid disorders associated with the five EQ-5D-5L health dimensions (Table 5). Locations of the recovery residences were included as possible confounders in each regression model. The likelihood ratio test was performed to compare the goodness of fit between models in which residences were nested within the city where they lived (Austin, El Paso, Houston, Midland, and San Angelo). The observed differences were not statistically significant; thus, residence locations were excluded from the final models. No imputation was done for missing data. We used a critical level of ≤ 0.05 for statistical significance in the regression models. All analyses were performed using Stata/MP16 (45).

**Table 1:** Resident's characteristics associated with EQ-5D-5L health dimensions (*n* = 358).

**Participant's characteristics**	**Total *n* (%)**	**Mobility**		**Self-care**		**Usual activities**		**Pain/discomfort**		**Anxiety/depression**	
		**No problems 291 (82.0)**	**Any problem 64 (18.0)**	**No problems 336 (94.6)**	**Any problem 19 (5.4)**	**No problems 307 (86.5)**	**Any problem 48 (13.5)**	**No problems 166 (46.8)**	**Any problem 189 (53.2)**	**No problems 86 (24.2)**	**Any problem 269 (75.8)**
Age (Mean [SD])	36.0 [8.9]	**35.0 [8.1]**	**40.6 [11.0]**	35.9 [8.6]	39.1 [13.2]	**35.6 [8.6]**	**38.9 [10.5]**	**34.6 [7.3]**	**37.3 [9.9]**	36.2 [9.4]	36.0 [8.8]
**Race-ethnicity**											
Non-Hispanic white	246 (68.7)	205 (70.4)	38 (59.4)	230 (68.4)	13 (68.4)	212 (69.1)	31 (64.6)	116 (69.9)	127 (67.2)	61 (70.9)	182 (67.7)
Racial-ethnic minority	112 (31.3)	86 (29.6)	26 (40.6)	106 (31.6)	6 (31.6)	95 (30.9)	17 (35.4)	50 (30.1)	62 (32.8)	25 (29.1)	87 (32.3)
**Sex**											
Male	213 (59.7)	177 (61.0)	35 (54.7)	201 (60.0)	11 (57.9)	188 (61.4)	24 (50.0)	**111 (66.9)**	**101 (53.7)**	58 (67.4)	154 (57.5)
Female	144 (40.3)	113 (39.0)	29 (45.3)	134 (40.0)	8 (42.1)	118 (38.6)	24 (50.0)	**55 (33.1)**	**87 (46.3)**	28 (32.6)	114 (42.5)
**Education**											
< HS/HS diploma	176 (49.4)	144 (49.8)	32 (50.0)	167 (50.0)	9 (47.4)	**157 (51.5)**	**19 (39.6)**	84 (50.9)	92 (48.9)	42 (49.4)	134 (50.0)
Some college/Voc.Tech.	152 (42.7)	120 (41.5)	30 (46.9)	140 (41.9)	10 (52.6)	**121 (39.7)**	**29 (60.4)**	64 (38.8)	86 (45.7)	35 (41.2)	115 (42.9)
Bachelor's degree/higher	28 (7.9)	25 (8.6)	2 (3.1)	27 (8.1)	0 (0.0)	**27 (8.8)**	**0 (0.0)**	17 (10.3)	10 (5.3)	8 (9.4)	19 (7.1)
**Employment status**											
Unemployed	236 (66.3)	**183 (63.3)**	**50 (78.1)**	218 (65.1)	15 (83.3)	**195 (63.7)**	**38 (80.8)**	**99 (60.0)**	**134 (71.3)**	**48 (55.8)**	**185 (69.3)**
Employed	120 (33.7)	**106 (36.7)**	**14 (21.9)**	117 (34.9)	3 (16.7)	**111 (36.3)**	**9 (19.2)**	**66 (40.0)**	**54 (28.7)**	**38 (44.2)**	**82 (30.7)**
**Marital status**											
Single/never married	221 (61.9)	188 (64.8)	32 (50.0)	**213 (63.6)**	**7 (36.8)**	197 (64.4)	23 (47.9)	112 (67.9)	108 (57.1)	55 (64.0)	165 (61.6)
Married/common-law/committed	43 (12.0)	33 (11.4)	10 (15.6)	**38 (11.3)**	**5 (26.3)**	35 (11.4)	8 (16.7)	18 (10.9)	25 (13.2)	9 (10.5)	34 (12.7)
Separated/divorced/widowed	93 (26.1)	69 (23.8)	22 (34.4)	**84 (25.1)**	**7 (36.8)**	74 (24.2)	17 (35.4)	35 (21.1)	56 (29.6)	22 (25.6)	69 (25.7)
**Hazardous drinking**											
No or low risk/	325 (90.8)	268 (92.1)	56 (87.5)	307 (91.4)	17 (89.5)	**284 (92.5)**	**40 (83.3)**	156 (94.0)	168 (88.9)	**85 (98.8)**	**239 (88.9)**
High risk	33 (9.2)	23 (7.9)	8 (12.5)	29 (8.6)	2 (10.5)	**23 (7.5)**	**8 (16.7)**	10 (6.0)	21 (11.1)	**1 (1.2)**	**30 (11.1)**
**Tobacco use**											
No	29 (8.1)	22 (7.6)	7 (10.9)	27 (8.0)	2 (10.5)	**25 (8.1)**	**4 (8.3)**	14 (8.4)	15 (7.9)	**4 (4.7)**	**25 (9.3)**
Yes	329 (91.9)	269 (92.4)	57 (89.1)	309 (92.0)	17 (89.5)	**282 (91.9)**	**44 (91.7)**	152 (91.6)	174 (92.1)	**82 (95.3)**	**244 (90.7)**
**Number of illicit drugs used**											
0	88 (24.6)	**76 (26.1)**	**11 (17.2)**	83 (24.7)	4 (21.0)	81 (26.4)	6 (12.5)	50 (30.1)	37 (19.6)	27 (31.4)	60 (22.3)
1	38 (10.6)	**24 (8.3)**	**14 (21.9)**	37 (11.0)	1 (5.3)	33 (10.8)	5 (10.4)	19 (11.4)	19 (10.0)	10 (11.6)	28 (10.4)
2	62 (17.3)	**50 (17.2)**	**12 (18.7)**	56 (16.7)	6 (31.6)	51 (16.6)	11 (22.9)	30 (18.1)	32 (16.9)	18 (20.9)	44 (16.4)
3+	170 (47.5)	**141 (48.4)**	**27 (42.2)**	160 (47.6)	8 (42.1)	142 (46.2)	26 (54.2)	67 (40.4)	101 (53.4)	31 (36.1)	137 (50.9)
Satisfaction with health (Mean [SD])	2.7 [1.0]	**2.8 [0.9]**	**2.1 [1.2]**	**2.7 [1.0]**	**1.8 [1.2]**	**2.8 [0.9]**	**2.0 [1.2]**	**3.0 [0.8]**	**2.4 [1.0]**	**3.2 [0.8]**	**2.5 [1.0]**
**MOUD duration**											
< 1 year	105 (29.3)	89 (30.6)	16 (25.0)	**104 (30.9)**	**1 (5.3)**	**98 (31.9)**	**7 (14.6)**	55 (33.1)	50 (26.4)	33 (38.4)	72 (26.8)
1–5 years	240 (67.0)	192 (66.0)	45 (70.3)	**219 (65.2)**	**18 (94.7)**	**198 (64.5)**	**39 (81.2)**	104 (62.7)	133 (70.4)	50 (58.1)	187 (69.5)
>5 years	13 (3.6)	10 (3.4)	3 (4.7)	**13 (3.9)**	**0 (0.0)**	**11 (3.6)**	**2 (4.2)**	7 (4.2)	6 (3.2)	3 (3.5)	10 (3.7)
MOUD duration (Mean [SD])	1.2 [1.7]	**1.1 [0.1]**	**1.7 [2.7]**	1.2 [1.7]	1.5 [0.2]	**1.1 [1.4]**	**1.8 [2.8]**	1.2 [1.4]	1.3 [1.9]	1.0 [1.3]	1.3 [1.8]

HS, High school. Voc. Tech., Vocational/Technical diploma. Differences in counts result from missing data. Bolded values indicate statistical significance at p < 0.05.

**Table 2:** Comorbid diagnoses by self-reported EQ-5D-5L health dimensions (*n* = 358).

**Comorbidities**	**Total n (%)**	**Mobility**		**Self-care**		**Usual activities**		**Pain/discomfort**		**Anxiety/depression**	
		**No problems 291 (82.0)**	**Any problem 64 (18.0)**	**No problems 336 (94.6)**	**Any problem 19 (5.4)**	**No problems 307 (86.5)**	**Any problem 48 (13.5)**	**No problems 166 (46.8)**	**Any problem 189 (53.2)**	**No problems 86 (24.2)**	**Any problem 269 (75.8)**
**Mental health disorders**											
No	263 (73.5)	209 (71.8)	51 (79.7)	**242 (72.0)**	**18 (94.7)**	**218 (71.0)**	**42 (87.5)**	115 (69.3)	145 (76.7)	**54 (62.8)**	**206 (76.6)**
Yes	95 (26.5)	82 (28.2)	13 (20.3)	**94 (28.0)**	**1 (5.3)**	**89 (29.0)**	**6 (12.5)**	51 (30.7)	44 (23.3)	**32 (37.2)**	**63 (23.4)**
**Respiratory disorders**											
No	264 (73.7)	**222 (76.3)**	**40 (62.5)**	**252 (75.0)**	**10 (52.6)**	**235 (76.5)**	**27 (56.2)**	130 (78.3)	132 (69.8)	**72 (83.7)**	**190 (70.6)**
Yes	94 (26.3)	**69 (23.7)**	**24 (37.5)**	**84 (25.0)**	**9 (47.4)**	**72 (23.5)**	**21 (43.8)**	36 (21.7)	57 (30.2)	**14 (16.3)**	**79 (29.4)**
**Neurological disorders**											
No	289 (80.7)	**241 (82.8)**	**45 (70.3)**	272 (81.0)	14 (73.7)	**253 (82.4)**	**33 (68.8)**	**150 (90.4)**	**136 (72.0)**	**78 (90.7)**	**208 (77.3)**
Yes	69 (19.3)	**50 (17.2)**	**19 (29.7)**	64 (19.0)	5 (26.3)	**54 (17.6)**	**15 (31.2)**	**16 (9.6)**	**53 (28.0)**	**8 (9.3)**	**61 (22.7)**
**Cardiovascular disorders**											
No	293 (81.8)	**245 (84.2)**	**46 (71.9)**	278 (82.)	13 (68.4)	**260 (84.7)**	**31 (64.6)**	**147 (88.5)**	**144 (76.2)**	74 (86.0)	217 (80.7)
Yes	65 (18.2)	**46 (15.8)**	**18 (28.1)**	58 (17.3)	6 (31.6)	**47 (15.3)**	**17 (35.4)**	**19 (11.5)**	**45 (23.8)**	12 (14.0)	52 (19.3)
**Endocrine disorders**											
No	334 (93.3)	**277 (95.2)**	**55 (85.9)**	316 (94.0)	16 (84.2)	**291 (94.8)**	**41 (85.4)**	**161 (97.0)**	**171 (90.5)**	82 (95.3)	250 (92.4)
Yes	24 (6.7)	**14 (4.8)**	**9 (14.1)**	20 (6.0)	3 (15.8)	**16 (5.2)**	**7 (14.6)**	**5 (3.0)**	**18 (9.5)**	4 (4.7)	19 (7.1)
**Musculoskeletal disorders**											
No	297 (83.0)	**250 (85.9)**	**44 (68.7)**	281 (83.6)	13 (68.4)	**261 (85.0)**	**33 (68.7)**	**153 (92.2)**	**141 (74.6)**	73 (84.9)	221 (82.8)
Yes	61 (17.0)	**41 (14.1)**	**20 (31.3)**	55 (16.4)	6 (31.6)	**46 (15.0)**	**15 (31.3)**	**13 (7.8)**	**48 (25.4)**	13 (15.1)	48 (17.8)
**Digestive disorders**											
No	328 (93.3)	270 (92.8)	56 (87.5)	309 (92.0)	17 (89.5)	284 (92.5)	42 (87.5)	156 (94.0)	170 (90.0)	83 (96.5)	243 (90.3)
Yes	30 (6.7)	21 (7.2)	8 (12.5)	27 (8.0)	2 (10.5)	23 (7.5)	6 (12.5)	10 (6.0)	19 (10.0)	3 (3.5)	26 (9.7)
**Urogenital disorders**											
No	334 (93.3)	274 (94.2)	57 (89.1)	315 (93.8)	16 (84.2)	**290 (94.5)**	**41 (85.4)**	157 (94.6)	174 (92.1)	83 (96.5)	248 (92.2)
Yes	24 (6.7)	17 (5.8)	7 (10.9)	21 (6.2)	3 (15.8)	**17 (5.5)**	**7 (14.6)**	9 (5.4)	15 (7.9)	3 (3.5)	21 (7.8)
**Other comorbidities**											
No	302 (84.4)	**252 (86.6)**	**47 (73.4)**	285 (84.8)	14 (73.7)	**264 (86.0)**	**35 (72.9)**	148 (89.2)	151 (79.9)	72 (83.7)	227 (83.4)
Yes	56 (15.6)	**39 (13.4)**	**17 (26.6)**	51 (15.2)	5 (26.3)	**43 (14.0)**	**13 (27.1)**	18 (10.8)	38 (20.1)	14 (16.3)	42 (15.6)
**No. of comorbidities**											
0	96 (26.8)	**84 (28.9)**	**10 (15.6)**	90 (26.8)	4 (21.1)	**87 (28.3)**	**7 (14.6)**	**59 (35.5)**	**35 (18.5)**	**24 (27.9)**	**70 (26.0)**
1	121 (33.8)	**105 (36.1)**	**16 (25.0)**	117 (34.8)	4 (21.1)	**108 (35.2)**	**13 (27.0)**	**61 (36.7)**	**60 (31.7)**	**38 (44.2)**	**83 (30.9)**
2	74 (20.7)	**59 (20.3)**	**15 (23.4)**	71 (21.1)	3 (15.7)	**66 (21.5)**	**8 (16.7)**	**31 (18.7)**	**43 (22.8)**	**15 (17.4)**	**59 (21.9)**
3 or more	67 (18.7)	**43 (14.8)**	**23 (35.9)**	58 (17.3)	8 (42.1)	**46 (15.0)**	**20 (41.7)**	**15 (9.0)**	**51 (27.0)**	**9 (10.5)**	**57 (21.2)**

Differences in counts result from missing data. Bolded values indicate statistical significance at p < 0.05.

**Table 3:** Resident's characteristics associated with comorbid disorders (*n* = 358).

**Resident's characteristics**	**Total n (%)**	**Mental disorder 95 (26.5)**	**Respiratory disorder 94 (26.3)**	**Neurological disorder 69 (19.3)**	**Cardio-vascular 65 (18.2)**	**Endocrine disorder 24 (6.7)**	**Musculo-skeletal 61 (17.0)**	**Digestive disorder 30 (8.4)**	**Urogenital disorder 24 (6.7)**	**Other comorbidities 56 (15.6)**
Age (Mean [SD])	36.0 [8.9]	35.4 [8.3]	36.8 [9.9]	36.6 [9.3]	**41.0 [10.2]**	38.3 [9.3]	**38.8 [9.0]**	**40.1 [11.3]**	36.5 [5.7]	37.0 [9.7]
**Race-ethnicity**										
Non-Hispanic White	246 (68.7)	59 (62.1)	**75 (79.8)**	54 (78.3)	43 (66.2)	**12 (50.0)**	48 (78.7)	24 (80.0)	**21 (87.5)**	35 (62.5)
Racial-ethnic minority	112 (31.3)	36 (37.9)	**19 (20.2)**	15 (21.7)	22 (33.8)	**12 (50.0)**	13 (21.3)	6 (20.0)	**3 (12.5)**	21 (37.5)
**Sex**										
Male	213 (59.7)	56 (59.0)	51 (54.3)	**28 (41.2)**	37 (56.9)	**6 (25.0)**	30 (50.0)	16 (53.3)	**5 (21.7)**	**15 (27.3)**
Female	144 (40.3)	39 (41.0)	43 (45.7)	**40 (58.8)**	28 (43.1)	**18 (75.0)**	30 (50.0)	14 (46.7)	**18 (78.3)**	**40 (72.7)**
**Education**										
< HS/HS diploma	176 (49.4)	54 (57.5)	43 (46.2)	**25 (36.8)**	29 (45.3)	10 (43.5)	23 (38.3)	11 (37.9)	9 (37.5)	26 (47.3)
Some college/Voc. Tech.	152 (42.7)	34 (36.2)	42 (45.2)	**37 (54.4)**	29 (45.3)	13 (56.5)	32 (53.3)	16 (55.2)	15 (62.5)	25 (45.4)
Bachelor's degree/higher	28 (7.9)	6 (6.4)	8 (8.6)	**6 (8.8)**	6 (9.4)	0 (0.0)	5 (8.3)	2 (6.9)	0 (0.0)	4 (7.3)
**Employment status**										
Unemployed	236 (66.3)	60 (63.2)	60 (64.5)	51 (75.0)	47 (73.4)	19 (82.6)	43 (71.7)	20 (69.0)	17 (70.8)	41 (73.2)
Employed	120 (33.7)	35 (36.8)	33 (35.5)	17 (25.0)	17 (26.6)	4 (17.4)	17 (28.3)	9 (31.0)	7 (29.2)	15 (26.8)
**Marital status**										
Single/Never married	221 (61.9)	67 (71.3)	50 (53.2)	36 (52.2)	**26 (40.0)**	**9 (37.5)**	31 (50.8)	13 (43.3)	10 (41.7)	29 (51.8)
Married/Common-law/Committed	43 (12.0)	8 (8.5)	12 (12.8)	8 (11.6)	**12 (18.5)**	**6 (25.0)**	10 (16.4)	6 (20.0)	6 (25.0)	8 (14.3)
Separated/Divorced/Widowed	93 (26.1)	19 (20.2)	32 (34.0)	25 (36.2)	**27 (41.5)**	**9 (37.5)**	20 (32.98)	11 (36.7)	8 (33.3)	19 (33.9)
**Hazardous drinking**										
No/low risk	325 (90.8)	86 (90.5)	82 (87.2)	62 (89.9)	**53 (81.5)**	22 (91.7)	55 (90.2)	26 (86.7)	22 (91.7)	51 (91.1)
High risk	33 (9.2)	9 (9.5)	12 (12.8)	7 (10.1)	**12 (18.5)**	2 (8.3)	6 (9.8)	4 (13.3)	2 (8.3)	5 (8.9)
**Tobacco use**										
No	29 (8.1)	5 (5.3)	7 (7.5)	5 (7.3)	9 (13.8)	3 (12.5)	5 (8.2)	2 (6.7)	0 (0.0)	5 (8.9)
Yes	329 (91.9)	90 (94.7)	87 (92.5)	64 (92.7)	56 (86.2)	21 (87.5)	56 (91.8)	28 (93.3)	24 (100.0)	51 (91.1)
**Polysubstance use**										
No	88 (24.6)	**31 (32.6)**	23 (24.5)	16 (23.2)	**7 (10.8)**	6 (25.0)	13 (21.3)	6 (20.0)	7 (29.2)	18 (32.1)
Yes	270 (75.4)	**64 (67.4)**	71 (75.5)	53 (76.8)	**58 (89.2)**	18 (75.0)	48 (78.7)	24 (80.0)	17 (70.8)	38 (67.9)
Satisfaction with health (mean [SD])	2.7 [1.0]	**2.9 [0.8]**	**2.5 [0.1]**	2.6 [1.1]	**2.4 [1.1]**	2.6 [1.1]	**2.4 [1.0]**	**2.2 [1.1]**	2.3 [1.0]	**2.4 [1.1]**
**MOUD duration**										
< 1 year	105 (29.3)	**80 (84.2)**	20 (21.3)	13 (18.8)	14 (21.5)	8 (33.3)	**9 (14.8)**	5 (16.7)	3 (12.5)	11 (19.6)
1–5 years	240 (67.0)	**10 (10.5)**	72 (76.6)	54 (78.3)	49 (75.4)	13 (54.2)	**49 (80.3)**	24 (80.0)	21 (87.5)	42 (75.0)
>5 years	13 (3.6)	**5 (5.3)**	2 (2.1)	2 (2.9)	2 (3.1)	3 (12.5)	**3 (4.9)**	1 (3.3)	0 (0.0)	3 (5.4)
MOUD duration (Mean [SD])	1.2 [1.7]	**0.3 [1.1]**	1.3 [2.1]	1.4 [1.7]	1.6 [2.5**]**	1.7 [2.1]	**1.7 [2.6]**	1.6 [2.2]	1.3 [0.8]	1.4 [1.5]

Age = 19–72 years. Racial-ethnic minorities: Non-Hispanic Black or African American = 20 (5.6%), Non-Hispanic Other = 9 (2.5%), Hispanic = 83 (23.2%). HS, High school. Voc. Tech., Vocational/Technical diploma. Differences in counts result from missing data. Bolded values indicate statistical significance at p < 0.05.

**Table 4:** Multivariable logistic regression of resident's characteristics by comorbid disorders.

**Participant's characteristics**	**aOR (95% CI)**
**Mental health disorders**	
Polysubstance use (vs. no polysubstance use)	0.54 (0.28; 1.04)
Satisfaction with health (0–4)	1.30 (0.96; 1.77)
MOUD duration (0–19 years)	**0.14 (0.08; 0.22)**
**Respiratory disorders**	
Racial-Ethnic minorities (vs. non-Hispanic White)	**0.46 (0.26; 0.81)**
Satisfaction with health (0–4)	**0.77 (0.60; 0.98)**
**Neurological disorders**	
Female (vs. Male)	**2.44 (1.41; 4.21)**
Some college/Voc./College (vs. < High school/High school diploma)	**1.78 (1.02; 3.10)**
**Cardiovascular disorders**	
Age (years)	**1.06 (1.03; 1.10)**
Married/Common-law/Committed (vs. Single/Never married)	**2.47 (1.07; 5.67)**
Separated/Divorced/Widowed (vs. Single/Never married)	**2.01 (1.03; 3.93)**
High risk for Hazardous drinking (vs. No/Low risk)	**2.86 (1.25; 6.59)**
Polysubstance use (vs. No polysubstance use)	**3.40 (1.36; 8.52)**
**Endocrine disorders**	
Female (vs. Male)	**5.03 (1.87; 13.5)**
Racial-Ethnic minorities (vs. non-Hispanic White)	**2.66 (1.10; 6.39)**
Married/Common-law/Committed (vs. Single/Never married)	**4.80 (1.51; 15.26)**
Separated/Divorced/Widowed (vs. Single/Never married)	2.15 (0.79; 5.85)
**Musculoskeletal disorders**	
Age (years)	**1.03 (1.00; 1.06)**
Satisfaction with health (0–4)	**0.75 (0.57; 0.99)**
MOUD duration (0–19 years)	1.10 (0.94; 1.29)
**Digestive disorders**	
Age (years)	**1.04 (1.00; 1.08)**
Satisfaction with health (0–4)	**0.68 (0.47; 0.98)**
**Urogenital disorders**	
Female (vs. Male)	**6.27 (2.25; 17.41)**
Racial-Ethnic minorities (vs. non-Hispanic White)	**0.28 (0.08; 0.97)**
**Other comorbid disorders**	
Female (vs. Male)	**5.17 (2.68; 9.69)**
Satisfaction with health (0–4)	**0.76 (0.06; 0.37)**

Bolded values indicate statistical significance at p < 0.05.

**Table 5:** Logistic regression models to predict EQ-5D-5L health dimensions.

**A) Univariate regression models**					
	**Mobility**	**Self-care**	**Usual activities**	**Pain/discomfort**	**Anxiety/depression**
	**OR (95% CI)**	**aOR (95% CI)**	**OR (95% CI)**	**OR (95% CI)**	**OR (95% CI)**
Mental health disorders (vs. none)	—	0.14 (0.02; 1.09)	**0.35 (0.14; 0.85)**	—	**0.56 (0.31; 0.87)**
Respiratory disorders (vs. none)	**1.94 (1.09; 3.43)**	**2.7 (1.06; 6.87)**	**2.54 (1.35; 4.76)**	—	**2.14 (1.14; 4.01)**
Neurological disorders (vs. none)	**2.04 (1.10; 3.77)**	—	**2.13 (1.08; 4.19)**	**3.65 (1.99; 6.69)**	**2.86 (1.31; 6.25)**
Cardiovascular disorders (vs. none)	**2.08 (1.11; 3.91)**	—	**3.03 (1.56; 5.92)**	**2.42 (1.35; 4.33)**	—
Endocrine disorders (vs. none)	**3.24 (1.33; 7.85)**	—	**3.11 (1.21; 8.00)**	**3.39 (1.23; 9.34)**	—
Musculoskeletal disorders (vs. none)	**2.77 (1.49; 5.17)**	—	**2.58 (1.30; 5.12)**	**4.01 (2.08; 7.71)**	—
Urogenital disorders (vs. none)	—	—	**2.91 (1.14; 7.45)**	—	—
Other comorbid disorders (vs. none)	**2.33 (1.22; 4.47)**	—	**2.28 (1.12; 4.65)**	—	—
Number of comorbidities (vs. none)					
1 comorbidity (vs. none)	1.28 (0.55; 2.97)	—	1.50 (0.57; 3.91)	1.66 (0.96; 2.87)	0.75 (0.41; 1.37)
2+ comorbidities (vs. none)	**3.13 (1.47; 6.65)**	—	**3.10 (1.30; 7.45)**	**3.44 (1.99; 5.95)**	1.66 (0.87; 3.14)
**B) Covariate-Adjusted Multivariable Regression Models**					
	**Mobility**	**Self-Care**	**Usual Activities**	**Pain/Discomfort**	**Anxiety/Depression** ^*^
	**aOR (95% CI)**	**aOR (95% CI)**	**aOR (95% CI)**	**aOR (95% CI)**	**aOR (95% CI)**
Age (years)	**1.05 (1.01; 1.09)**	—	1.01 (0.97; 1.05)	**1.02 (1.00; 1.05)**	—
Female (vs. Male)	—	—	—	1.50 (0.91; 2.45)	—
Some college/Voc.Tech/College (vs. < High school/High school diploma)	—	—	1.52 (0.76; 3.06)	—	—
Employed (vs. Unemployed)	0.71 (0.35; 1.44)	—	0.51 (0.22; 1.18)	0.75 (0.45; 1.23)	**0.59 (0.34; 0.99)**
Married/Common-law/Committed (vs. Single/Never married)	—	2.65 (0.75; 9.41)	—	—	—
Separated/Divorced/Widowed (vs. Single/Never married)	—	1.63 (0.52; 5.09)	—	—	—
High risk for hazardous drinking (Vs. Low risk)	—	—	1.48 (0.49; 4.48)	—	—
Used 1 illicit drug (vs. No illicit drug use)	**3.36 (1.20; 9.45)**	—	—	—	—
Used 2+ illicit drugs (vs. No illicit drug use)	1.15 (0.52; 2.57)	—	—	—	—
Satisfaction with health (0–4)	**0.55 (0.41; 0.75)**	**0.53 (0.34; 0.82)**	**0.57 (0.42; 0.80)**	**0.50 (0.38; 0.66)**	**0.41 (0.29; 0.59)**
MOUD duration (years)	1.05 (0.87; 1.27)	—	1.11 (0.89; 1.37)	—	—
Mental health disorders (vs. none)	—	0.23 (0.03; 1.80)	0.69 (0.25; 1.87)	—	0.66 (0.38, 1.15)
Respiratory disorders (vs. none)	1.33 (0.66; 2.71)	1.85 (0.68; 5.03)	1.86 (0.87; 3.99)	—	1.70 (0.85; 3.40)
Neurological disorders (vs. none)	1.52 (0.71; 3.27)	—	1.00 (0.43; 2.35)	**2.71 (1.38; 5.33)**	2.19 (0.94; 5.13)
Cardiovascular disorders (vs. none)	0.94 (0.41; 2.14)	—	1.78 (0.75; 4.23)	1.63 (0.82; 3.24)	—
Endocrine disorders (vs. none)	2.28 (0.75; 6.90)	—	1.69 (0.53; 5.35)	1.29 (0.40; 4.17)	—
Musculoskeletal disorders (vs. none)	1.46 (0.68; 3.12)	—	1.25 (0.54; 2.91)	**2.57 (1.25; 5.29)**	—
Urogenital disorders (vs. none)	—	—	1.81 (0.61; 5.43)	—	—
Other comorbid disorders (vs. none)	1.41 (0.63; 3.11)	—	1.39 (0.58; 3.30)	—	—

Bolded values indicate statistical significance at p < 0.05. ^*^Hazardous drinking was excluded from the analyses due to the small cell count. Used 1 or 2+ illicit drugs refers to the number of different illicit substances used by residents in the past 3 months and does not take into account the duration beyond 3 months or frequency of use.

## 3 Results

### 3.1 Sociodemographic characteristics

Three hundred and fifty-eight residents, ages 19–72 years (mean [SD] = 36.0 [8.9]), were included in the analyses. In Table 1, demographic characteristics contributing to differences across several HRQoL dimensions were age, sex, education, employment status, marital status, hazardous drinking, tobacco use, number of illicit drugs used, as well as satisfaction with health, and MOUD duration. Most residents had a high school diploma or less (49.4%), were non-Hispanic White (68.7%), male (59.7%), unemployed (66.3%), single/never married (61.9%), engaged in polysubstance use (75.4%), and reported taking MOUD for 1–5 years (67.0%). The overall mean [SD] satisfaction with health score was 2.7 [1.0]. Most residents reported anxiety/depression problems (75.8%), followed by pain/discomfort (53.2%), mobility (18.0%), usual activities (13.5%), and self-care (5.4%) problems.

### 3.2 Comorbid mental and physical health disorders

Table 2 describes comorbidities and their associations with HRQoL dimensions. All comorbid disorders, except digestive disorders, contributed to differences observed across all the HRQoL dimensions. The most frequently reported comorbidities were mental health disorders (26.5%), followed by respiratory (26.3%), neurological (19.3%), cardiovascular (18.2%), musculoskeletal (17.0%), other comorbid (15.6%), digestive (8.4%), endocrine (6.7%), and urogenital (6.7%) disorders. Most residents reported at least one comorbidity (73.2%). The highest number of comorbid disorders observed in any HRQoL dimension was in the usual activities dimension (*n* = 8), followed by mobility (*n* = 6), pain/discomfort (*n* = 4), anxiety/depression (*n* = 3), and self-care (*n* = 2). The number of comorbidities residents reported was associated with all the dimensions except self-care.

### 3.3 Associations between resident's sociodemographic characteristics and comorbid disorders

In Table 3, resident's characteristics contributing to differences across each comorbid disorder were age, race/ethnicity, sex, education, marital status, hazardous drinking, polysubstance use, satisfaction with health, and MOUD duration. Most residents with mental health disorders reported engaging in polysubstance use (67.4%) and taking MOUD for less than a year (84.2%). 79.8% of individuals with a respiratory disorder were non-Hispanic White. More than half of the residents with neurological disorders were female (58.8%) and had attended some college or had a vocational/technical diploma (54.4%). The mean age [SD] of residents with cardiovascular disorder was 41.0 [10.2], musculoskeletal disorder was 38.8 [9.0], and digestive disorder was 40.1 [11.3]. Most residents with cardiovascular disorders were separated, divorced, or widowed (41.5%), engaged in polysubstance use (89.2%), and had no or low risk for hazardous drinking (81.5%). Among residents with an endocrine disorder, 75.0% were female, 37.5% were single or never married, and 37.5% were separated, divorced, or widowed. For residents with a musculoskeletal disorder, the majority had been taking MOUD for 1–5 years, with a mean health satisfaction score of 2.4 [1.0]. Most residents with an urogenital disorder were non-Hispanic White (87.5%) and female (78.3%).

Table 4 summarizes the multivariable logistic regression of resident's characteristics by comorbid disorders. Less time spent taking MOUD was associated with mental health disorders (aOR = 0.14; 95% CI = 0.08–0.22). Racial and ethnic minority residents were less likely to report respiratory disorders (aOR = 0.46; 95% CI = 0.26–0.81) and urogenital (aOR = 0.28; 95% CI = 0.08–0.97) disorders but more likely to report endocrine disorders (aOR = 2.66; 95% CI = 1.10–6.39) than non–Hispanic White residents. Female residents were more likely to report neurological (aOR = 2.44; 95% CI = 1.41–4.21), endocrine (aOR = 5.03; 95% CI = 1.87–13.5), urogenital (aOR = 6.27; 95% CI = 2.25–17.41), and other comorbid disorders (aOR = 5.17; 95% CI = 2.68–9.69) than male residents.

Residents who attended some college or had a vocational/technical diploma or college degree (vs. less than high school or high school diploma) were more likely to report neurological disorders (aOR = 1.78; 95% CI = 1.02–3.10). Increasing age was positively associated with cardiovascular (aOR = 1.06; 95% CI = 1.03–1.10), musculoskeletal (aOR = 1.03; 95% CI = 1.00–1.06), and digestive (aOR = 1.04; 95% CI = 1.00–1.08) disorders. Residents who engaged in hazardous drinking (aOR = 2.86; 95% CI = 1.25–6.59) and polysubstance use (aOR = 3.40; 95% CI = 1.36–8.52) were more likely to report cardiovascular disorders than individuals who did not engage in hazardous drinking or polysubstance use. Married residents or those in a common–law marriage or committed relationship were more likely to report cardiovascular (aOR = 2.47; 95% CI = 1.07–5.67) and endocrine (aOR = 4.80; 95% CI = 1.51–15.26) disorders than those who were single or never married.

### 3.4 Associations between resident's sociodemographic characteristics, comorbid disorders, and EQ-5D-5L HRQoL dimensions

#### 3.4.1 Mobility dimension

Table 5A summarizes the univariate regression models predicting comorbidities. Respiratory (OR = 1.94; 95% CI = 1.09–3.43), neurological (OR = 2.04; 95% CI = 1.10–3.77), cardiovascular (OR = 2.08; 95% CI = 1.11–3.91), endocrine, (OR = 3.24; 95% CI = 1.33–7.85), musculoskeletal (OR = 2.77; 95% CI = 1.49–5.17), and other comorbid (OR = 2.33; 95% CI = 1.22–4.47) disorders were positively associated with mobility problems. Residents with two or more comorbidities were more likely to report mobility problems (OR = 3.13; 95% CI = 1.47–6.65) than residents without comorbidities. In the covariate–adjusted regression model (Table 5B), increasing age (aOR = 1.05; 95% CI = 1.01–1.09) and illicit drug use in the past 3 months (aOR = 3.36; 95% CI = 1.20–9.45) were positively associated with mobility problems. Residents with lower health satisfaction scores were more likely to report mobility problems (aOR = 0.55; 95% CI = 0.41–0.75) than those with higher scores.

#### 3.4.2 Self–care dimension

In Table 5A, respiratory disorders were positively associated with self–care problems (OR = 2.70; 95% CI = 1.06–6.87). In Table 5B, residents with lower health satisfaction scores were more likely to report problems conducting self–care (aOR = 0.53; 95% CI = 0.34–0.82) than those with higher satisfaction scores.

#### 3.4.3 Usual activities dimension

In Table 5A, respiratory (OR = 2.54; 95% CI = 1.35–4.76), neurological (OR = 2.13; 95% CI = 1.08–4.19), cardiovascular (OR = 3.03; 95% CI = 1.56–5.92), endocrine (OR = 3.11; 95% CI = 1.21–8.00), musculoskeletal (OR = 2.58; 95% CI = 1.30–5.12), urogenital (OR = 2.91; 95% CI = 1.14–7.45), and other comorbid (OR = 2.28; 95% CI = 1.12–4.65) disorders and having two or more comorbidities (OR = 3.10; 95% CI = 1.30–7.45) were positively associated with problems conducting usual activities. Residents with mental health disorders were less likely to report problems conducting usual activities (OR = 0.35; 95% CI = 0.14–0.85). In Table 5B, residents with lower health satisfaction scores were more likely to report problems conducting usual activities (aOR = 0.57; 95% CI = 0.42–0.80) than those with higher scores.

#### 3.4.4 Pain/discomfort dimension

In Table 5A, neurological (OR = 3.65; 95% CI = 1.99–6.69), cardiovascular (OR = 2.42; 95% CI = 1.35–4.33), endocrine (OR = 3.39; 95% CI = 1.23–9.34), and musculoskeletal (OR = 4.01; 95% CI = 2.08–7.71) disorders and having two or more comorbidities (OR = 3.44; 95% CI = 1.99–5.95) were positively associated with pain/discomfort problems. In Table 5B, increasing age (aOR = 1.02; 95% CI = 1.00–1.05), neurological disorders (aOR = 2.71; 95% CI = 1.38–5.33), and musculoskeletal disorders (aOR = 2.57; 95% CI = 1.25–5.29) were positively associated with pain/discomfort problems. Residents with lower health satisfaction scores were more likely to report pain/discomfort problems (aOR = 0.50; 95% CI = 0.38–0.66) than those with higher satisfaction scores.

#### 3.4.5 Anxiety/depression dimension

In Table 5A, respiratory (OR = 2.14; 95% CI = 1.14–4.01) and neurological (OR = 2.86; 95% CI = 1.31–6.25) disorders were positively associated with anxiety/depression problems, while mental health disorders were negatively associated with anxiety/depression problems (OR = 0.56; 95% CI = 0.31–0.87). In Table 5B, employed residents were less likely to report anxiety/depression problems (aOR = 0.59; 95% CI = 0.34–0.99). Residents with lower health satisfaction scores were more likely to report anxiety/depression problems (aOR = 0.41; 95% CI = 0.29–0.59) than those with higher satisfaction scores.

## 4 Discussion

This study is the first to examine associations between participants' characteristics, 36 comorbid diagnoses classified as mental health disorders or based on their associations with seven major body systems, and five EQ-5D-5L HRQoL dimensions (frequencies of reported problems) among recovery residents taking MOUD in the US. Research has demonstrated that individuals seeking treatment for SUDs have lower HRQoL than individuals without SUDs or comorbidities (6). Our findings also indicate that most individuals seeking recovery support for SUDs in recovery residences have substantial comorbidities and problems influencing their HRQoL, indicating significant impairment in their overall health and functioning (see Supplementary Table 2 for a list of leading OUD comorbidities).

Participants in our sample were relatively young (mean age = 36.0); thus, it is surprising that the majority presented with a comorbid diagnosis, which along with their SUD affects their HRQoL. In the unadjusted regression model, the usual activities dimension was the most affected by comorbidities, followed by mobility and pain/discomfort dimensions. Individuals with mental health disorders were less likely to report problems affecting their HRQoL (usual activities or anxiety/depression dimensions). The high rates of comorbidities among a primarily young sample and their impact on HRQoL and healthcare costs highlight the need for integrated care models, including screening and individualized treatment of comorbidities and OUD, to improve overall health and recovery outcomes.

Existing integrated care models provide concurrent treatment for substance use and mental health disorders and are effective in decreasing substance use and symptoms of mental illness (46). A systematic review of the literature on integrated care for concurrent mental health and substance use disorders indicates that an integrated approach produces better outcomes and is more cost-effective for treating comorbid disorders than standard care (46). Therefore, these integrated care models should be expanded to incorporate the treatment of physical comorbid disorders to improve HRQoL and recovery and health outcomes.

Treating comorbid disorders in residential care settings can significantly lower the cost of treating comorbidities and improve outcomes, including reducing medical inpatient and outpatient healthcare utilization (47, 48). Studies have demonstrated the effectiveness of recovery housing in decreasing substance use and incarceration rates, improving abstinence and mental health, and increasing education and employment rates and monthly income (39, 49, 50). Furthermore, several level IV recovery residences have demonstrated that their services can be expanded to provide therapeutically effective and cost-effective treatment for comorbid SUDs among special populations, including individuals living with HIV/AIDS, mental illness, and previously incarcerated inmates leaving prison treatment (51). Thus, recovery residences provide an ideal integrated care setting for monitoring and treating SUD and medical comorbidities, particularly as they have evolved into diverse models to meet the changing needs of individuals with SUD, including persons taking MOUD.

We found that certain sociodemographic factors and mental health predicted residents' HRQoL. For instance, increasing age was significantly associated with poorer HRQoL, particularly the mobility and pain/discomfort dimensions. While long-term opioid use could be contributing to these comorbidities, it is also possible that pre-existing mobility problems, especially those associated with pain or negative affect, may have contributed to the continued use of opioids as a means of managing symptoms (16–20, 22). Mental health disorders were the most frequently reported comorbidity. The anxiety/depression dimension had the highest proportion of residents reporting HRQoL problems. Our findings are consistent with research that demonstrated that opioid- and mental health-related symptoms and disorders frequently co-occur, and mental health disorders are risk factors for opioid misuse and OUD (52, 53).

Neurological and musculoskeletal disorders ranked among the most frequently reported comorbid disorders. Residents with neurological and musculoskeletal disorders had higher odds of reporting pain/discomfort problems. The negative impact of neurological and musculoskeletal disorders, such as arthritis, chronic pain, epilepsy, and migraine, on HRQoL is well-documented (54–57). A common pathway to developing OUD stems from treating chronic pain. Integrated care approaches should target OUD and neurological and musculoskeletal disorders to improve overall health, functioning, and recovery outcomes. Policymakers should support researchers in developing evidence-based interventions for chronic pain to decrease prescription opioid use and improve residents' response to OUD treatment and recovery. Recovery residences should integrate comprehensive pain treatment programs or link residents to appropriate pain management programs, including non-pharmacological therapy and treatment for comorbidities.

Most residents reported polysubstance use in the past 3 months, and illicit drug use in the past 3 months was associated with mobility problems. Illicit drug use can alter motor skills and cause mobility impairment (58–63). OUD prevention, treatment, and recovery programs should prioritize the treatment of and recovery from polysubstance use to improve recovery outcomes, overall health, and HRQoL. This is crucial as drug-related overdose deaths, including methamphetamine, benzodiazepine, and cocaine overdose deaths, are rising with or without opioid use (31, 64).

Research indicates that HRQoL is high at MOUD initiation and within the first few months to 1 year (65, 66). However, HRQoL was not associated with MOUD duration in our sample, suggesting that several factors, such as having comorbidities and lower levels of recovery capital (67), influence HRQoL beyond OUD treatment, abstinence, or decreases in opioid use. Thus, focusing only on pharmacological treatments of OUD may have a limited influence on HRQoL. Residents should be provided an integrated care model for comorbidities and SUDs, along with other recovery support, including building new recovery capital and fortifying existing recovery capital, which are essential at different stages of recovery (67–69).

Several clinical, research, policy, and practice implications are noteworthy. First, physical and mental comorbidities frequently occur with OUD and are associated with significant impairment of functioning and wellbeing (4, 6, 9). This enhanced need for healthcare may exacerbate healthcare spending and disparities in treatment and recovery outcomes. Policymakers should support an interprofessional model of care that integrates treatments for comorbidities and OUD with recovery support services. Researchers need to identify all predictors of HRQoL, including comorbidities, assess longitudinal changes in HRQoL, and develop evidence-based interventions and strategies for clinical practice and integration with recovery residences to address these predictors and improve HRQoL. With improved knowledge and understanding, policymakers, health planners, recovery residence administrators/operators, and clinicians can plan and deliver more effective, person-centered, integrated treatment and recovery support. Furthermore, recovery residence operators should be trained in assessing HRQoL, comorbidities, and other predictors of HRQoL. This will enable them to link residents to the appropriate clinical care and support services to improve their health and functioning.

Finally, a systematic review and meta-analysis of 58 cohort studies among individuals who use opioids found that although opioid-related overdose was the most common cause of death, deaths related to the liver, cancer, trauma (e.g., accidents, injury, and poisoning), and cerebrovascular, digestive, cardiovascular, respiratory, and nervous systems were also common (26). The authors also reported that individuals in treatment for OUD and taking MOUD had lower mortality rates than those out of treatment (26). Given the significant mortality risk among individuals with opioid dependency and the high rates of mental and physical comorbidities in our sample, we must prioritize efforts aimed at preventing untimely deaths through the provision of integrated care among recovery residents with OUD and other SUDs, using recovery residences as the primary setting for integrated care models. By fostering collaborations between multidisciplinary teams, including primary care and behavioral health providers, recovery residences have the potential to provide a holistic, efficient, and non-stigmatizing way to enhance access to care and effectively manage OUD and mental and physical comorbidities.

Our analyses have several limitations. First, we did not collect information on comorbidity treatment and opioid use duration. Additional treatment/care residents received and long-term opioid use may have impacted their HRQOL. Second, we cannot determine temporal links between HRQoL and factors we found influenced HRQoL due to analyses of baseline data. These factors may be premorbid to OUD and increase residents' risk of OUD, secondary to OUD, or develop with OUD. Third, 36 diseases were collapsed and classified as mental health disorders or based on their associations with seven major body systems. Variance in the associations observed may be attributed to only one comorbidity. Our findings should be replicated with longitudinal studies with larger sample sizes to assess for significant associations between specific diseases and HRQoL. Furthermore, our study is prone to several biases, including selection bias due to a sample selection that may not reflect the characteristics of individuals in other recovery residences and social desirability and recall biases due to our reliance on self-reported data. Finally, residents were predominantly non-Hispanic White, male, and residing in 14 levels II and III recovery residences in five Texas cities, limiting the generalizability of our findings.

## 5 Conclusions

Most residents reported substantial comorbidities and problems affecting their HRQoL. There were sociodemographic differences in comorbidities and HRQoL. Our findings highlight the need to consider multiple contextual factors when examining HRQoL, including comorbid mental and physical health disorders and recovery capital. The treatment of and recovery from OUD is complex and is further complicated by existing comorbidities, which may have a greater impact on HRQoL than SUD itself in this population. This underscores the importance of integrated care models that address both SUDs and comorbidities to improve overall quality of life. Recovery residences are supportive communities that can be leveraged to implement short-term, integrative, comprehensive recovery and healthcare service models to enhance residents' access to clinical treatment and recovery support. This may reduce the severity of complications associated with comorbidities and SUDs and facilitate the achievement and maintenance of recovery.

## Data Availability

The datasets presented in this article are not readily available because the dataset is not publicly available due to restrictions in research permission and to protect participants' identities. Requests to access the datasets should be directed to johnny.m.wilkerson@uth.tmc.edu.
