# Supplementary material for: Characteristics and quality of life of people living with comorbid disorders in substance use recovery residences
Source: Front Public Health. 2024 Nov 19;12:1412934. doi: 10.3389/fpubh.2024.1412934 (PMC11611819; doi:10.3389/fpubh.2024.1412934)
Supplement: Supplementary file 1 [file Table_1.docx]

Supplementary Table 1. Self-reported 5Q-5D-5L problems by comorbidities (n = 358)

| **5Q-5D-5L dimensions** | **Total** | **No Comorbidities** | **Comorbidities** |
| --- | --- | --- | --- |
|  | **n (%)** | **n (%)** | **n (%)** |
| **Mobility** |  |  |  |
| No problems | 291 (81.3) | 84 (87.5) | 207 (79.0) |
| Slight problems | 40 (11.2) | 7 (7.3) | 33 (12.6) |
| Moderate problems | 14 (3.9) | 3 (3.1) | 11 (4.2) |
| Severe problems | 10 (2.8) | 0 (0.0) | 10 (3.8) |
| Unable to walk | — | — | — |
| **Self-Care** |  |  |  |
| No problems | 336 (93.8) | 90 (93.8) | 246 (93.9) |
| Slight problems | 10 (2.8) | 3 (3.1) | 7 (2.7) |
| Moderate problems | 8 (2.3) | 1 (1.0) | 7 (2.7) |
| Severe problems | 1 (0.8) | 0 (0.0) | 1 (0.4) |
| Unable to wash or dress | — | — | — |
| **Usual Activities** |  |  |  |
| No problems | 307 (85.8) | 87 (90.6) | 220 (84.0) |
| Slight problems | 29 (8.1) | 4 (4.2) | 25 (9.5) |
| Moderate problems | 14 (3.9) | 3 (3.1) | 11 (4.2) |
| Severe problems | 4 (1.1) | 0 (0.0) | 4 (1.5) |
| Unable to do | 1 (0.3) | 0 (0.0) | 1 (0.4) |
| **Pain/Discomfort** |  |  |  |
| No pain | 166 (46.4) | 59 (61.5) | 107 (40.8) |
| Slight pain | 72 (20.1) | 17 (17.7) | 55 (21.0) |
| Moderate pain | 78 (21.8) | 15 (15.6) | 63 (24.1) |
| Severe pain | 26 (7.3) | 2 (2.1) | 24 (9.2) |
| Extreme pain | 13 (3.6) | 1 (1.0) | 12 (4.6) |
| **Anxiety/Depression** |  |  |  |
| Not anxious or depressed | 86 (24.0) | 24 (25.0) | 62 (23.7) |
| Slightly | 105 (29.3) | 35 (36.5) | 70 (26.7) |
| Moderately | 104 (29.1) | 23 (24.0) | 81 (30.9) |
| Severely | 35 (9.8) | 7 (7.3) | 28 (10.7) |
| Extremely | 25 (7.0) | 5 (5.2) | 20 (7.6) |

_Note: Differences in counts result from missing data._

Supplementary Table 2. Comorbid disorders by primary body system association

| **Comorbidities** | **Number (%)** |
| --- | --- |
| 1. **Mental health disorders** | **95 (26.5%)** |
| Depression - Persistent depressive disorder (PDD), major depressive disorder (MDD), Seasonal affective disorder (SAD) | 53 (14.8) |
| Anxiety - panic, generalized, and social anxiety disorders, specific phobias | 51 (14.3) |
| Bipolar disorder | 30 (8.4) |
| Post-traumatic stress disorder (PTSD) | 24 (6.7) |
| Obsessive-compulsive disorder (OCD) | 6 (1.7) |
| Schizophrenia, Schizoaffective, and other psychosis-related disorders | 4 (1.1) |
| Other | 6 (1.7) |
| 1. **Respiratory disorders** | **94 (26.3%)** |
| Asthma | 41 (11.5) |
| Bronchitis | 39 (10.9) |
| Pneumonia | 34 (9.5) |
| Frequent respiratory infections | 8 (2.2) |
| Tuberculosis (TB) | 7 (2.0) |
| Emphysema | 4 (1.1) |
| Other | 7 (2.0) |
| 1. **Neurological disorders** | **69 (19.3%)** |
| Migraines | 44 (12.4) |
| Memory loss | 33 (9.2) |
| Epilepsy | 4 (1.1) |
| Other | 3 (0.8) |
| 1. **Cardiovascular disorders** | **65 (18.2%)** |
| High blood pressure | 56 (15.6) |
| High cholesterol | 11 (3.1) |
| Stroke | 4 (1.1) |
| Heart disease | 2 (0.6) |
| 1. **Musculoskeletal disorders** | **61 (17.0%)** |
| Bone fractures | 33 (9.2) |
| Arthritis | 28 (7.8) |
| Osteoporosis | 6 (1.7) |
| Chronic pain | 3 (0.8) |
| Other | 2 (0.6) |
| 1. **Digestive disorders** | **30 (8.4%)** |
| Ulcer | 16 (4.5) |
| Gallbladder disease | 10 (2.8) |
| Cirrhosis | 4 (1.1) |
| Other | 2 (0.6) |
| 1. **Endocrine disorders** | **24 (6.7%)** |
| Thyroid trouble | 11 (3.1) |
| Type II diabetes | 8 (2.2) |
| Type I diabetes | 5 (1.4) |
| 1. **Urogenital disorders** | **24 (6.7%)** |
| Urinary Tract Infections (UTI) | 18 (5.0) |
| Prostate trouble | 3 (0.8) |
| Other | 4 (1.1) |
| 1. **Other comorbidities** | **56 (15.6%)** |
| Anemia | 34 (9.5) |
| Cancer | 10 (2.8) |
| Hepatitis A, B, and C | 7 (2.0) |
| Other | 10 (2.8) |

_Note: Differences in counts result from missing data._

Supplementary Table 3. Alcohol, tobacco, and illicit drug use in the past 90 days

| **Substances** | **n (%)** |
| --- | --- |
| Alcohol | 344 (96.1) |
| Tobacco - E-cigarettes, cigarettes, smoking tobaccos (e.g., pipes, cigars, cigarillos, and little filtered cigars), chewing tobacco, snuff, and dip | 329 (91.9) |
| Street opioids (e.g., heroin, opium, fentanyl, etc) | 197 (55.0) |
| Methamphetamine | 151 (42.2) |
| Benzodiazepines (e.g., Diazepam (Valium), Alprazolam (Xanax))* | 139 (38.8) |
| Marijuana (Cannabis, pot, hash, etc) | 134 (37.5) |
| Prescription opioids (e.g., morphine, methadone, codeine, oxycodone) * | 93 (26.0) |
| Cocaine (Coke, crack, etc) | 87 (24.3) |
| CBD (cannabidiol) products | 38 (10.6) |
| Prescription stimulants (e.g., Ritalin, Concerta, Dexedrine, Adderall, diet pills) * | 36 (10.1) |
| Kratom | 35 (9.8) |
| MDMA (e.g., Ecstasy) | 23 (6.4) |
| Hallucinogens (e.g., LSD, acid, mushrooms) | 23 (6.4) |
| Cough syrup mixed with soda (e.g., purple drank, sizzurp) | 20 (5.6) |
| Non-prescription GHB (e.g.,Grievous Bodily Harm, Liquid Ecstasy, Georgia) | 17 (4.8) |
| Synthetic marijuana | 12 (3.4) |
| PCP (e.g., Angel dust, Rocket fuel) | 11 (3.1) |
| Amphetamines (e.g., Uppers) | 10 (2.8) |
| Ketamine (Special K or Vitamin K) | 9 (2.5) |
| Barbiturates (e.g., Mephobarbital (Mebacut), pentobarbital sodium (Nembutal)) * | 8 (2.2) |
| Inhalants (e.g., snappers, whippets, glue, gas, paint thinner) | 7 (2.0) |
| Sexual enhancement inhalants (e.g., Poppers, rush, nitrous oxide) | 4 (1.1) |

_Note: Differences in counts result from missing data. *Misuse of prescription medications, such as opioids, stimulants, sedatives or sleeping pills, and barbiturates_
